# Depression in Myotonic Dystrophy type 1: clinical and neuronal correlates

**DOI:** 10.1186/1744-9081-6-25

**Published:** 2010-05-19

**Authors:** Stefan Winblad, Christer Jensen, Jan-Eric Månsson, Lena Samuelsson, Christopher Lindberg

**Affiliations:** 1 Neuromuscular Centre, Department of Neurology, Sahlgrenska University Hospital, Göteborg, Sweden; 2 Department of Psychology, Göteborg University, Sweden; 3 Department of Radiology, Sahlgrenska University Hospital, Göteborg, Sweden; 4 Institute of Neuroscience and Physiology, Sahlgrenska Academy, Göteborg University, Sweden; 5 Department of Clinical Genetics, Sahlgrenska University Hospital, Göteborg, Sweden

## Abstract

**Background:**

This study was designed to investigate the prevalence and correlates of depression in Myotonic dystrophy type 1 (DM1).

**Methods:**

Thirty-one patients with DM1 and 47 subjects in a clinical contrast group, consisting of other neuromuscular disorders, including Spinal muscular atrophy, Limb girdle muscle atrophy and Facioscapulohumeral dystrophy, completed Beck Depression Inventory (BDI). We aimed to establish whether different factors associated with DM1 correlated with ratings in the BDI.

**Results:**

Signs of a clinical depression were prevalent in 32% of the patients with DM1, which was comparable with ratings in the clinical contrast group. The depressive condition was mild to moderate in both groups. In DM1, a longer duration of clinical symptoms was associated with lower scores on the BDI and higher educational levels were correlated with higher scores on depression. We also found a negative association with brain white matter lesions.

**Conclusions:**

Findings indicate significantly more DM1 patients than normative collectives showing signs of a clinical depression. The depressive condition is however mild to moderate and data indicate that the need for intervention is at hand preferentially early during the disease process.

## Background

Depression is an important health issue because of its high lifetime prevalence and association with substantial disability [1]. An increased risk of having major depression is associated with chronic disease, neurological and neuromuscular disorder and a comorbid state of depression has been found to incrementally worsen health [1-3]. Without treatment, depression has a tendency to assume a chronic course, be recurrent and over time to be associated with increasing disability [1]. Consequently, the possible comorbidity of depression with chronic disease is important to acknowledge.

Myotonic dystrophy type 1 (DM1) is a progressive, dominantly inherited, multisystem disease caused by an expanded and unstable trinucleotide CTG repeat localized to the 3' untranslated region of the dystrophia myotonica protein kinase (DMPK) gene on chromosome 19q13.3 [4]. The expansion of CTG repeats causes muscle wasting, myotonia, heart conduction defect, lens cataract, endocrine dysfunction and brain abnormalities through different molecular mechanisms [5]. A growing body of evidence shows that a RNA gain-of-function mechanism plays a major role in the disease development but the process explaining neurocognitive dysfunction is however more uncertain [6-8]. It is known that DM1 is a disorder associated with neuropsychological deficits, including reduced attention, speed, visuoconstructive and executive abilities [9]. Accompanying brain abnormalities is predominant in frontal and temporal, occipital and subcortical regions, including both cerebral atrophy and white matter hyperintense lesions [7]. Mood and personality disorders associated with apathy and social avoidance is recurrently seen [7,10]. Some authors have found significantly higher prevalence of depression as compared to healthy controls [11,12] but others have found no indication of major depression [13-15]. Furthermore, the cause of depression in DM1 is still not clear, where some authors have tried to explain depression as a psychological adaptation to the disease [11] and others, as a direct manifestation of genetic and/or CNS abnormalities [13,14]. However, to date, no study has confirmed any significant association between depression and clinical and/or biological parameters. The aims of this study were to characterize and to evaluate occurrence of depression in patients with DM1 and to explore clinical, genetic and neurocognitive correlates.

## Methods

### Subjects

Demographic and clinical data are presented in Table 1. Sixty adult patients with genetically confirmed DM1, associated with the Neuromuscular Centre, Sahlgrenska University Hospital in Göteborg, were invited to participate. Inclusion criteria were: age 18-65 years and no history of major psychiatric or somatic illness, major brain injury or alcohol misuse. Twenty-nine declined, mainly due to non-compliance in taking part in the brain imaging and CSF-measurement, leaving 31 patients with adult onset/classical DM1. Forty-seven patients with other neuromuscular disorders formed a clinical contrast group. This group comprised 13 patients with spinal muscular atrophy, 14 with limb girdle muscle atrophy and 20 with facioscapulohumeral dystrophy. In DM1 patient's strength in handgrip was measured with the Grippit instrument [16] and the overall muscle function was scored using the Muscle Impairment Rating Scale (MIRS) [17]. In both groups, Brooke's grading system of mobility was scored [18]. All participants gave informed and written consent and the medical ethics committee at the Sahlgrenska Academy, Göteborg University, approved the study.

**Table 1:** Demographic and clinical description of subjects

	DM1 (n = 31)	Contrast group (n = 47)
Age	41.8 (9.5, 23-58)	43.5 (15.0, 18-64)
Sex	14 M, 17 F	15 M, 32 F
Education (years)	10.9 (2.2, 8-18)	10.4 (1.8, 9-15)
Age at onset	26.2 (8.7, 15-50)	17 (14.5, 0-60)
Disease duration (years)	16.8 (10.7, 3-38)	27 (16, 5-60)
CTG-repeats	578 (401, 100-2000)	-
Fatigue*	25 (81%)	-
MIRS**	3.6 (0.8, 1-5)	-
Grip force^† ^(Newton)	104 (48, 20-144)	-
Brooke-rating^††^	0.5 (1.1)	5.9 (13.9)

* Patients scoring the presence of abnormal fatigue in a self-rating procedure described in Winblad et al, 2005 [9].

** Muscular Impairment Rating Scale [17]. This score ranges from 1 (no muscular impairment) to 5 (severe proximal weakness).

^† ^Scores on a handgrip force measurement using the Grippit instrument. Mean (SD) for healthy controls = 331 (77) Newton [16].

^†† ^Brookes functional test and grading system, zero implies normal function and higher values indicates increasing levels of immobility [18].

Data in Table 1 is presented as mean, standard deviation and range.

### Self-rating of depression

We used a Swedish version of Beck Depression Inventory (BDI), a widely used 21-item standardized self-report questionnaire measuring depression on a 4-point scale ranging from 0 to 3 [19,20]. Proposed cut-off scores may be interpreted as follows: 1-9, minimal depression; 10-16, mild depression; 17-29, moderate depression; 30-63, severe depression [20]. We also performed an item-analysis on two separable factors of depression in the BDI; a cognitive affective dimension (item 1-13) and a somatic dimension (item 14-21) [20].

### Neuropsychological assessment

All subjects participated in a neuropsychological investigation comprising tests measuring verbal ability (Vocabulary), verbal fluency (FAS), visual construction and memory ability (RCFT, Block design, Picture completion), verbal memory (RAVLT), speed (Trail Making Test, Digit symbol), attention (TMT, Digit span, Spatial span) and executive function (Stroop, TMT, FAS and WCST). Tests, references and procedure have been described in earlier studies by the authors [8,9].

### Brain MRI and cerebrospinal fluid examination

As to explore directly visible and indirect manifestations of brain functioning we performed a brain MRI examination and a measurement of cerebrospinal fluid (CSF) markers indicative of neuronal degeneration and amyloidogenesis.

Twenty-nine patients had MRI examinations which were performed on a 1,0 Tesla magnet (Siemens Magnetom Impact, Erlangen, Germany) or a 1,5 Tesla magnet (Siemens Symphony, Erlangen, Germany). The protocol consisted of a T2 and a proton density or FLAIR sequence in axial projection. A coronal FLAIR and a sagittal T2 sequence were included at the end of the study when the MRI equipment was updated. One experienced neuroradiologist blinded to clinical symptoms evaluated all MR images. A rating of white matter lesions [21,22], brain atrophy and ventricular size [23] was performed according to procedures earlier described in Winblad et al. 2008 [8].

Twelve ml of CSF was obtained by routine lumbar puncture, centrifuged and stored in aliquots at - 80°C. Analyses of CSF-tau levels were made using a sandwich ELISA (Innotest hTAU-Ag, Innogenetics, Gent, Belgium) constructed to measure both normal and phosphorylated tau. Quantification of tau phosphorylated at threonine 181 (P-tau) was performed with a sandwich ELISA procedure (Innotest Phospho-Tau (181P), Innogenetics, Gent, Belgium). Levels of Aβ42 were done using an ELISA (Innotest β-amyloid _(1-42)_, Innogenetics) [24,25].

### Genetic analysis

DNA was extracted from peripheral blood lymphocytes and analysed for expansion of the CTG repeat in the DMPK gene. The analysis was performed with PCR and southern blot using the probe pM10M6 [26]. The size of the CTG-expansions was assessed visually from exposed x-ray films.

### Statistical analysis

Data were analysed using SPSS base 11.5 (Chicago IL) and are presented as median and interquartile range (IR). Due to skewness in the data set, non-parametric statistics were used in comparisons between groups. The Spearman rank correlation test was used when analysing correlations between measures. Significance level was set at P < 0.05.

## Results

**Figure 1:**
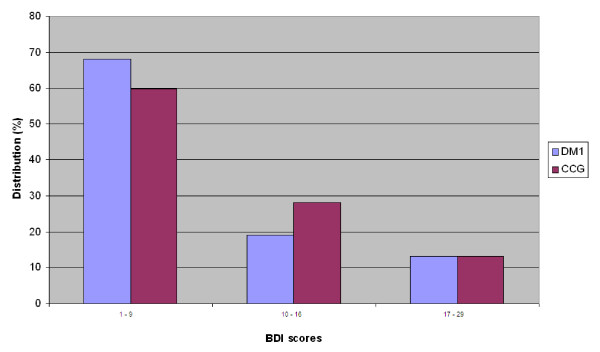
**Distribution of BDI scores of patients with DM1 and clinical contrast group patients**. Description of values: 1-9 = minimal depression, 10-16 = mild depression, 17-29 = moderate depression. Cut off value on clinical depression = 10 (20).

Results on the BDI rating showed that DM1 patients and the clinical contrast group (CCG) scored with a median value of 6 (IR = 5) and 7 (IR = 10), respectively. There was no significant difference between groups. Figure 1 shows the distribution of scores. Ratings above a cut off score for depression set at 10 [20] were found in 10 DM1 (32%) and 19

(40%) CCG patients. Among DM1 patients indicating clinical depression, a mild depression was most prevalent and signs of a moderate depression were found in a minority of patients. The same pattern was found in the CCG. Ratings indicating a severe depressive condition (BDI score > 30) were absent in both groups. When analysing the distribution on separate items in the BDI, high scores were found on the somatic dimension of depression associated with fatigability, hypersomnia and muscle weakness but also high scores on negative body image and somatic pre-occupation. In contrast, low scores were found on the cognitive-emotional dimension, including suicidal ideas, sadness, pessimism, guilt, self-dislike and punishment. A significant difference (P < 0.001) between somatic and social-cognitive dimensions was found in both the DM1 (Z = -3.32) and the CCG (Z = -3.55).

**Figure 2:**
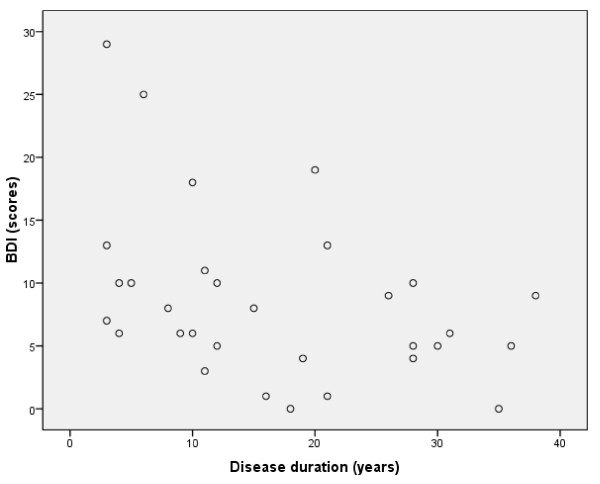
**Correlation between DM1 patient's scores on Beck Depression Inventory (BDI) and duration of disease (years), *r*_s _= -.426, P < 0.02**.

Disease duration correlated significantly with BDI scores (*r*_s _= -.426, P < 0.02) (Figure 2) indicating that a clinical depression was typically present in patients with disease duration less than 20 years. This association was not found in the CCG.

We did not find any difference on BDI scores when comparing four groups of DM1 patients with different CTG repeat expansion sizes (50-150, 151-500, 501-1000 and > 1000 repeats, respectively). No significant association was found between BDI ratings and CSF levels of Aβ42 and Tau. However, we found a significant difference between groups (Z = -2.3, P < 0.02) on BDI scores when comparing patients with white matter lesions (Median BDI score = 6) and patients without (Median BDI score = 8.7). When analyzing the difference between four groups, based on grading of white matter lesion load, a pattern emerged, indicating that mild-few punctuate foci or patchy non-confluent lesion to moderate-beginning confluent loci was gradually more associated with lower BDI scores than a condition associated with the absence of white matter lesions. No difference was found on other brain imaging data including the presence/absence of atrophy, periventricular lesions and ventricular widening. Ventricular brain ratio did not correlate with BDI scores.

Concerning neuropsychological measures, gender, age, age of onset, MIRS score, handgrip strength or ratings of fatigue, there was no association with BDI-scores. However a positive correlation was found between years of education and ratings on depression (*r*_s _= .44, P < 0.02) indicating that DM1 patients with higher education (> 11 years) also scored higher on the BDI.

## Discussion

The present study identified clinical depression in 32% of the patients with DM1. The depressive condition was mild to moderate and comparable to ratings in a clinical contrast group comprising other neuromuscular disorders. The point prevalence of depression in normative collectives ranges between 2-5% [27], indicating a significantly higher prevalence of depression among DM1 patients and the CCG. When analysing single items on the BDI, a large majority of patients scored high on a somatic dimension and contrastingly low on cognitive-emotional content. Consequently, few DM1 patients meet a psychiatric criterion of depression due to low scores on the emotional-cognitive dimension. This reasonably explains why measurement using a direct psychiatric evaluation (DSM-IV) [28] has not confirmed major depression as significant in DM1 [12,13]. When comparing criteria for depression [28] with DM1 specific bodily symptoms, such as fatigue, muscle weakness and sleep disorder, there is a considerable overlap, indicating that high scores on the BDI were mainly associated with an impact of the disease, rather than a psychological reaction on the condition. These findings reasonably have clinical importance, when considering that the high prevalence of fatigue, reduced initiative and facial weakness in DM1, may lead to a misconception of a major depressive condition, comparable to misjudgements done in other neurological disorders [29].

Data showed that depression was associated with earlier stages of DM1. This finding has been highlighted in other diseases [30] but stands in sharp contrast to assumptions postulating that the progressive nature of the disease contribute to the depressive condition [11]. Indeed, our findings may seem contra-intuitive when considering that DM1 is a progressive disabling disease [11]. Findings in the present study may be explained by patients use of instrumental coping strategies as to meet symptoms and consequences late during the disease in contrast with predominantly emotion-focused coping (crisis reaction and depression) early on. Low scores on depression, late in the disease, may also be associated with a narrowing of life expectancies and demands as the disease progress, leading to reduced stress and negative emotional reactions. A large difference between expected life circumstances and the actual life-situation might contribute to signs of depression early in the course of the disease, with closeness to an imagined life without DM1. A discrepancy between expected and actual life circumstances may also contribute to the prevalence of depression among patients with higher education. Notably however, correlations between BDI-ratings, disease duration and education were not found in the CCG, indicating disease specific associations in DM1.

Findings in this study showed significantly lower BDI scores in DM1 patients with white matter lesions (WMLs) as compared to patients with an absence of WMLs. This indicates that brain damage may actually "protect" against depression - a finding comparable to an association described in a recent study on the prevalence of post-traumatic stress disorder and brain damage among combat veterans [31]. Hypothetically, this association may be explained by a clinical phenomenon described in DM1: a lack of awareness of deficit [7]. A lack of awareness may be associated with reduced levels of depression as the disease progress, due to the fact that a *reaction *to the condition reasonably involves *awareness *of the condition. Generally, the ability to interpret signals from the body but also to experience deficits have been associated with localised lesions [32]. These brain areas mainly include the prefrontal- and parieto-temporal cortex, insula and thalamus. Interestingly, when analyzing the localisation of lesions in respect to brain areas in DM1 we found a main localisation in corresponding areas; central temporal and frontal areas, but also the insula region (data not shown). We also found an association, indicating a trend (P = 0.075) between lower BDI scores and lesions in the temporal cortex. These associations indicate the need for further studies on lack of awareness in DM1, including prevalence, causative factors and further consequences.

## Limitations

Conclusions based on present findings are limited by several factors. Due to used study design, i.e., the inclusion of only patients with adult onset DM1, it is still an open question if an onset < 10 years is associated with a higher prevalence of depression. Furthermore, the validity of used measurement of fatigue is reasonably limited. It is of importance to incorporate reliable measures on both fatigue and daytime sleepiness in future studies due to high prevalence and possible behavioural impact in DM1 [33]. An analysis of correlations between measures of depression and more sophisticated brain-imaging techniques such as PET and voxel based morphometry [34] may also reveal important associations between behavioural and imaging data beyond basic findings using conventional MRI.

## Conclusions

This study shows a clinical depressive condition in roughly one in three patients with DM1. This condition is mild to moderate and mainly associated with early stages of the disease, a higher educational level and the absence of brain white matter lesions. This indicates an impact of both clinical and social but also neuronal factors. Considering treatment options, our data indicate a need for intervention preferentially early in the disease process. Furthermore, depression in DM1 has to be understood in the context of factors directly associated with the disease, including apathy, reduced initiative and fatigue and reasonably at first hand targeting these factors, using both medication and proper behavioural and physical interventions [35,36].

## Competing interests

The authors declare that they have no competing interests.

## Authors' contributions

SW carried out the behavioural and neuropsychological examination and participated in the conception, design, analyses, interpretation and writing of the study. CJ carried out the brain imaging studies. J-EM carried out the cerebrospinal fluid analyses. LS carried out the molecular genetic studies. CL participated in the design, acquisition of data and helped to draft the manuscript. All authors read and approved the final manuscript.

## References

[B1] Moussavi S Chatterji S Verdes E Tandon A Patel V Ustun B Depression, chronic diseases, and decrements in health: results from the World Health Surveys Lancet 2007 370 851 58 10.1016/S0140-6736(07)61415-9 17826170

[B2] Benedetti F Bernasconi A Potiggia A Depression and neurological disorders Curr Opin Psychiatry 2006 19 14 18 10.1097/01.yco.0000194147.88647.7f 16612173

[B3] Kalkman JS Schillings ML Zwarts MJ van Engelen BGM Bleijenberg G Psychiatric disorders appear equally in patients with myotonic dystrophy, facioscapulohumeral dystrophy, and hereditary motor and sensory neuropathy type I Acta Neurol Scand 2007 115 265 70 10.1111/j.1600-0404.2006.00737.x 17376125

[B4] Meola G Myotonic dystrophies Curr Opin Neurol 2000 13 519 25 10.1097/00019052-200010000-00003 11073357

[B5] Cooper TA Chemical reversal of the RNA gain of function in myotonic dystrophy PNAS 2009 44 18433 34 10.1073/pnas.0910643106 PMC2774032 19864622

[B6] Wheeler TM Sobczak K Lueck JD Osborne RJ Lin X Dirksen RT Thornton CA Reversal of RNA dominance by displacement of protein sequestered on triplet repeat RNA Science 2009 325 336 39 10.1126/science.1173110 19608921 PMC4109973

[B7] Meola G Sansone V Cerebral involvement in myotonic dystrophies Muscle Nerve 2007 36 294 306 10.1002/mus.20800 17486579

[B8] Winblad S Månsson JE Blennow K Jensen C Samuelsson L Lindberg C Cerebrospinal fluid tau and amyloid beta42 protein in patients with myotonic dystrophy type 1 Eur J Neurol 2008 15 947 52 10.1111/j.1468-1331.2008.02217.x 18637827

[B9] Winblad S Lindberg C Hansen S Cognitive deficits and CTG repeat expansion size in classical myotonic dystrophy (DM1) Behav Brain Funct 2006 2 16 10.1186/1744-9081-2-16 16696870 PMC1475858

[B10] Winblad S Lindberg C Hansen S Temperament and character in patients with classical myotonic dystrophy type 1 (DM-1) Neuromuscul Disord 2005 15 287 92 10.1016/j.nmd.2004.12.003 15792867

[B11] Antonini G Soscia F Giubilei F De Carolis A Gragnani F Morinio S Health-related quality of life in myotonic dystrophy type 1 and its relationship with cognitive and emotional functioning J Rehabil Med 2006 38 181 85 10.1080/16501970500477967 16702085

[B12] Brumback RA Carlson KM The depression of myotonic dystrophy: response to imipramine J Neurol Neurosurg Psychiatry 1983 46 587 8 10.1136/jnnp.46.6.587 6875601 PMC1027462

[B13] Bungener C Jouvent R Delaporte C Psychopathological and emotional deficits in myotonic dystrophy J Neurol Neurosurg Psychiatry 1998 65 353 56 10.1136/jnnp.65.3.353 9728948 PMC2170247

[B14] Meola G Sansone V Perani D Scarone S Cappa S Dragoni C Executive dysfunction and avoidant personality trait in myotonic dystrophy type 1 (DM-1) and in proximal myotonic myopathy (PROMM/DM-2) Neuromuscul Disord 2003 13 813 21 10.1016/S0960-8966(03)00137-8 14678804

[B15] Duveneck MJ Portwood MM Wicks JJ Lieberman JS Depression in myotonic muscular dystrophy Arch Phys Med Rehabil 1986 67 875 77 3800615

[B16] Nordenskiöld U Grimby G Grip force in patients with rheumatoid arthtritis and fibromyalgia and in healthy subjects. A study with the Grippit instrument Scand J Rheumatol 1993 22 14 19 10.3109/03009749309095105 8434241

[B17] Mathieu J Boivin H Meunier D Gaudreault M Begin P Assessment of a disease-specific muscular impairment rating scale in myotonic dystrophy Neurol 2001 56 336 40 10.1212/wnl.56.3.336 11171898

[B18] Brooke MH A clinician's view of neuromuscular diseases 1986 2 Baltimore: Williams and Wilkinson

[B19] Beck AT Ward CH Mendelson M Mock J Erbaugh JK An inventory for measuring depression Arch Gen Psychiatry 1961 4 561 71 13688369 10.1001/archpsyc.1961.01710120031004

[B20] Beck AT Steer RA Beck Depression Inventory Manual-Swedish version. Psykologiförlaget. Fagernes, Norge 1996

[B21] Fazekas F Chawluk JB Alavi A Hurtig HI Zimmerman R MR signal abnormalities at 1.5 T in Alzheimer's dementia and normal aging Am J Roentgenol 1987 149 351 56 10.2214/ajr.149.2.351 3496763

[B22] Scheltens P Barkhof F Leys D Pruvo JP Nauta JJ Vermersch P A semiquantative rating scale for the assessment of signal hyperintensities on magnetic resonance imaging J Neurol Sci 1993 114 7 12 10.1016/0022-510X(93)90041-V 8433101

[B23] Di Costanzo A Di Salle F Santoro L Tessitore A Bonavita A Tedeschi G Pattern and significance of white matter abnormalities in myotonic dystrophy type 1: an MRI study J Neurol 2002 249 1175 1182 10.1007/s00415-002-0796-z 12242535

[B24] Sjögren M Vanderstichele H Ågren H Zachrisson O Edsbagge M Wickelso C Tau and Abeta42 in cerebrospinal fluid from healthy adults 21-93 years of age: establishment of reference values Clin Chem 2001 47 1776 81 11568086

[B25] Vanmechelen E Vanderstichele H Davidsson P Van Kerschaver E Perre B Van Der Sjögren M Quantification of tau phosphorylated at threonine 181 in human cerebrospinal fluid: a sandwich ELISA with a synthetic phosphopeptide for standardization Neurosci Lett 2000 285 49 52 10.1016/S0304-3940(00)01036-3 10788705

[B26] Brook JD McCurrach ME Harley HG Buckler AJ Church D Aburatani H Molecular basis of myotonic dystrophy: expansion of a trinucleotide (CTG) repeat at the 3'end of a transcript encoding a protein kinase family member Cell 1992 68 799 808 10.1016/0092-8674(92)90154-5 1310900

[B27] Gelder MG Lopéz J Ibor Jr Andreasen NC (ed) New Oxford textbook of psychiatry 2000 New York: Oxford University Press

[B28] Diagnostic and statistical manual of mental disorders DSM-IV-TR 2000 Fourth New York: American Psychiatric Publishing

[B29] Tickle-Degnen L Lyons KD Practitioners' impressions of patients with Parkinson's disease: the social ecology of the expressive mask Soc Sci Med 2004 58 603 14 10.1016/S0277-9536(03)00213-2 14652056

[B30] Brown R Jahanshahi M Depression in Parkinson's disease: a psychosocial viewpoint Adv Neurol 1995 65 61 84 7872153

[B31] Koenigs M Huey ED Raymont V Cheon B Solomon J Wassermann EM Focal brain damage protects against post-traumatic stress disorder in combat veterans Nat Neurosci 2008 11 232 37 10.1038/nn2032 18157125 PMC2693133

[B32] Orfei MD Robinson RG Bria P Caltagironi C Spalletta G Unawareness of illness in neuropsychiatric disorders: phenomenological certainty versus etiopathogenic vagueness Neuroscientist 2008 14 203 22 10.1177/1073858407309995 18057389

[B33] Orlikowski D Chevret S Quera-Salva MA Laforêt P Lofaso F Verschueren A Modafinil for the treatment of hypersomnia associated with myotonic muscular dystrophy in adults: a multicenter, prospective, randomized, double-blind, placebo-controlled, 4-week trial Clin Ther 2009 8 1765 73 10.1016/j.clinthera.2009.08.007 19808135

[B34] Kornblum C Reul J Kress W Grothe C Amanatidis N Klockgether T Cranial magnetic resonance imaging in genetically proven myotonic dystrophy type 1 and 2 J Neurol 2004 6 710 14 10.1007/s00415-004-0408-1 15311347

[B35] DeRubeis JR Siegle GJ Hollon SD Cognitive therapy versus medication for depression: treatment outcomes and neural mechanisms Nat Rev Neurosci 2008 9 788 96 10.1038/nrn2345 18784657 PMC2748674

[B36] Clark L Chamberlain SR Sahakian BJ Neurocognitive mechanisms in depression: implications for treatment Ann Rev Neurosci 2009 32 57 74 10.1146/annurev.neuro.31.060407.125618 19400725

